# Deciphering the molecular pathways disrupted in normal and neoplastic colonic tissue in inherited colorectal cancer syndromes

**DOI:** 10.1186/1897-4287-9-S1-O6

**Published:** 2011-03-10

**Authors:** Deborah W Neklason, Michelle W Done, Brett Milash, Lewis Frey, Nykole Sargent, Thérèse M Tuohy, Randall W Burt

**Affiliations:** 1 Department of Oncological Sciences, University of Utah, Salt Lake City, UT 84112-5550, USA; 2 Department of Biomedical Informatics, University of Utah, Salt Lake City, UT 84112-5550, USA; 3 Department of Medicine, University of Utah, Salt Lake City, UT 84112-5550, USA; 4 Huntsman Cancer Institute at University of Utah, Salt Lake City, UT 84112-5550, USA

## Background

Genetic profiling by RNA microarray of normal and neoplastic colonic tissue is used to identify biomarkers for development of a diagnostic tool for the inherited colon cancer syndromes. The utility of this method is to 1) properly classify patients where genetic diagnosis is incomplete 2) suggest molecular pathways that are shared between the syndromes and 3) identify molecular targets for prevention and treatment.

## Methods

RNA was extracted from fresh colonic biopsies obtained during endoscopy. Agilent 44K whole genome microarrays were run on 7-10 normal and neoplastic sample pairs from control, FAP, AFAP, Cowden syndrome, hyperplastic polyposis (HPP), and HNPCC (normal tissue only) patients. Microarray data were normalized, log-transformed, and analyzed for > 1.2 fold differential expression using GeneSifter software with a t-test adjusted by the Benjamini and Hochberg method. The most significant 100 mRNA probes were selected for evaluation. Select genes were confirmed by real-time quantitative PCR. Pathway analysis was done using prior knowledge resources including Genespring, KEGG and Cytoscape with microarray derived models.

## Results

The most significant 100 of 44,000 probes gave adjusted p-values < 3x10^-4^. Within this subset, the mRNAs from causative genes are decreased in normal colonic tissue from Cowden syndrome (*PTEN*), HNPCC (*MLH1*), and AFAP (*APC*) patients, but not from FAP (*APC*) patients, suggesting the presence of unstable mRNA and a molecular level of haploinsufficiency. The largest increased expression in HNPCC *vs.* controls included three genes with plekstrin homology domains (*TRIOBP*, *PLEKHF1*, *FGD1*), and two of these are also increased in Cowden syndrome (*PLEKHF1*, *FGD1*) along with *SMAD5*. There appear to be parallels between FAP and HPP. Normal HPP tissue has a decrease in *NKD2* which is a negative regulator of the canonical WNT signaling pathway. Normal FAP (*vs.* control) and HPP polyp (*vs.* normal) demonstrate a disruption in *MEKK4* (*MAP3K4*)*-ANXA2-PLG-MMP7* pathways. Additionally the proto-oncogene, *MET*, is up-regulated in FAP and AFAP normal as well as HPP polyp tissues. MET is a tyrosine kinase HGF receptor; mutations are associated with hereditary papillary renal carcinoma and it resides in a genetic region associated with common familial colon cancer. This gene may have a central role in predisposing cells for a proliferative phenotype as it interacts with beta-catenin pathway.

## Conclusions

Deciphering the molecular signatures of the inherited colon cancer syndromes has revealed, and will continue to reveal, interesting clues into pathways that are differentially regulated in normal colonic tissue, and those are altered during the neoplastic process.

## Disclosure and funding

There are no financial interests to disclose. This research was funded by NIH grants RC1-CA146329 (RWB); R03-CA150067 (DWN); R01-CA040641 (RWB) and Huntsman Cancer Foundation.

